# Anterior Chamber Flare as an Objective and Quantitative Noninvasive Method for Oculopathy in Transthyretin V30M Amyloidosis Patients

**DOI:** 10.1155/2018/3727543

**Published:** 2018-09-20

**Authors:** João Beirão, Vasco Miranda, Beatriz Pinheiro-Torres, João Coelho, Maria-João Menéres, Pedro Menéres

**Affiliations:** ^1^Instituto de Ciências Biomédicas Abel Salazar, Universidade do Porto, Porto, Portugal; ^2^Centro Hospitalar Porto, Porto, Portugal; ^3^Alimera Sciences, Alpharetta, USA

## Abstract

**Purpose:**

Assess the aqueous humor flare in transthyretin V30M amyloidosis patients (ATTRV30M).

**Materials and Methods:**

This is a retrospective, cross-sectional, noninterventional comparative study including 28 ATTRV30M patients with a unilateral scalloped iris. For comparative analysis, the fellow eye, the nonscalloped iris eye, from each patient was used as control. All patients underwent aqueous humor flare meter and intraocular pressure (IOP) measurements.

**Results:**

Mean aqueous humor flare was significantly higher in the eyes with the scalloped iris than the control group with the nonscalloped iris (14.1 ± 2.2 versus 6.5 ± 0.9 pc/ms, respectively). No significant differences in IOP were found in the scalloped iris eyes than those in the nonscalloped iris control group (17.1 ± 0.8 versus 16.8 ± 0.7 mmHg, respectively). No significant correlation was not found between the flare and the IOP value within groups.

**Conclusions:**

In this study, aqueous humor flare values in the scalloped iris eyes may be a valid marker for controlling the stage of the oculopathy in ATTRV30M patients.

## 1. Introduction

Hereditary transthyretin V30M amyloidosis (ATTRV30M) is an autosomal dominant disorder and is a very common form of hereditary amyloidosis caused by extracellular deposition of variants of transthyretin (TTR) in several tissues, including the eye [1, 2]. More than 100 amyloidogenic TTR mutations have been documented [3], but in the Portuguese type, the variant TTR has a substitution of methionine for valine at position 30. Despite the worldwide distribution of the disease, Portugal remains the main geographic focus of amyloidosis TTR V30M [4]. ATTRV30M patients are usually classified as presenting an early onset disease (onset before 50 years of age) or a late-onset disease (onset after 50 years of age). Early onset is associated with a more aggressive, rapidly progressing disease, especially if symptoms appear before 40 years of age [5]. Most Portuguese ATTRV30M patients are early-onset cases, with a worse prognosis regarding the severity of symptoms and an expected survival of 10 to 15 years without treatment. Liver transplantation is one of the treatments for ATTRV30M amyloidosis as it removes circulating mutant TTR and interrupts the progression of the disease and improve survival and quality of life [6]. Tafamidis, an oral, non-NSAID, highly specific TTR stabilizer has emerged as the new standard of care for ATTRV30M and remains the only medicine approved for the treatment of transthyretin amyloidosis in adult patients with stage 1 symptomatic polyneuropathy [7].

Nevertheless, the intraocular production of TTR V30M by the retinal and ciliary pigment epithelium remains unchanged, contributing to the progression of amyloid deposition-associated ocular manifestations such as abnormal conjunctival vessels, dry eye, scalloped pupils, deposition of amyloid on the anterior surface of the lens and on the pupil border, vitreous amyloidosis, glaucoma, and retinal angiopathy. Moreover, their prevalence increases over time [8].

Glaucoma is an ocular neurodegenerative disease and one of the most common causes of blindness worldwide [9]. It is characterized by retinal ganglion cell degeneration which can lead to optic atrophy and visual field defects [10]. Multiple factors play a role in the etiology and pathology of the disease, being intraocular pressure (IOP) the most important risk factor usually caused by abnormal aqueous humor outflow [11]. Extensive research over the last decade has shown that no amyloid secondary glaucomatous proteomics and oxidative damage can also lead to unfavourable effects on the trabecular meshwork and increased resistance to intraocular aqueous humor drainage [11–19]. Moreover, assessments of the involvement of ATTRV30M in ocular complications showed that glaucoma was strongly associated with the presence of scalloped iris [20]. Still, the nature of the interaction mechanism of iris deformation and aqueous humor flow was not evaluated and is not fully understood yet [8, 21].

However, the presence of scalloped iris before the onset of glaucoma was also observed [20]. Laser flare cell meter is an objective, sensitive, and noninvasive method for evaluating the aqueous flare and allows a quantitative assessment of anterior chamber inflammation and breakdown of the blood-aqueous barrier [22–24]. Several studies have shown altered flare values in glaucomatous eyes and speculated the role of the anterior chamber aqueous flare in predicting the risk of glaucoma [25–30]. Therefore, the aim of this analysis is to assess the blood-aqueous barrier (BAB) function with the use of a laser flare meter in ATTRV30M Portuguese patients with and without scalloped iris.

## 2. Materials and Methods

This was a retrospective, cross-sectional, noninterventional study conducted at the Ophthalmic Department from Centro Hospitalar do Porto, Porto. Written informed consent was obtained from all patients. This study was performed in accordance with the Declaration of Helsinki of the World Medical Association and was approved by the Ethics Committee of the Centro Hospitalar do Porto.

### 2.1. Patients

The study included a retrospective cohort of 28 consecutive patients (60.7% men; mean age: 46.5 (35–61) years) examined for ocular abnormalities at the Ophthalmology Service of Centro Hospitalar do Porto. All patients had the ATTR V30M mutation confirmed by genetic analysis and presented unilateral scalloped iris. These patients presented in both eyes no abnormalities in optic nerve and visual field nor ongoing treatment with ocular hypotensive eyedrops and/or previous glaucoma or ocular surgery. The only ocular treatment was artificial tears, and all eyes had best corrected visual acuity of 1.0 (Snellen equivalent). For comparative analysis, the fellow eye, the nonscalloped iris eye, from each patient was used as control. Data collection was from patients' medical records and included demographic data summarized in Table 1.

### 2.2. Measurement of Blood-Aqueous Barrier Function

To clarify the relationship between IOP increase and the blood-aqueous barrier function in both scalloped iris eyes and nonscalloped iris eyes (control), we measured the IOP values by using Goldmann applanation tonometry and the anterior chamber flare. Flare values were measured quantitatively by laser flare meter (LFM) (Kowa FM-700, Berkshire, UK) without pupil dilatation. On each occasion, 10 readings with a variation of less than 5% were taken. The highest and lowest values were discarded, and the remaining eight were averaged to obtain the flare measurement. Laser flare values were expressed in photon counts/millisecond (pc/ms) and standard deviation (SD) was calculated. Calibration of the laser flare meter was performed according to the manual.

### 2.3. Statistical Analysis

For continuous variables, such as IOP and anterior chamber flare, the observed values were summarized descriptively (mean ± SD). Student's two-group paired t-test was used to determine the significance of between-group differences in IOP and anterior chamber flare. Association between IOP and flare within each group was analyzed using Pearson's correlation coefficient. Statistical significance was declared at a type 1 error rate of 0.050. Statistical calculations and analyses were performed using SAS for PC, version 9.3 (SAS Inc, Cary, NC).

## 3. Results

**Figure 1:**
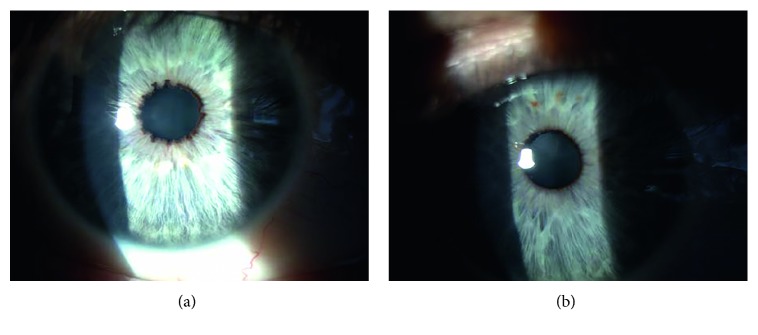
ATTRV30M scalloped iris eye (a) and the nonscalloped iris eye (control) (b) same patient.

**Figure 2:**
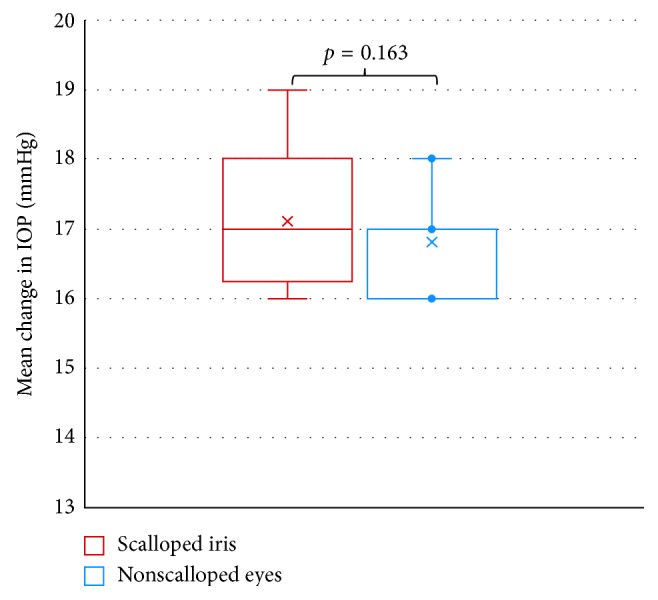
IOP distribution of ATTRV30M patients with scalloped iris and nonscalloped iris eyes (control).

**Figure 3:**
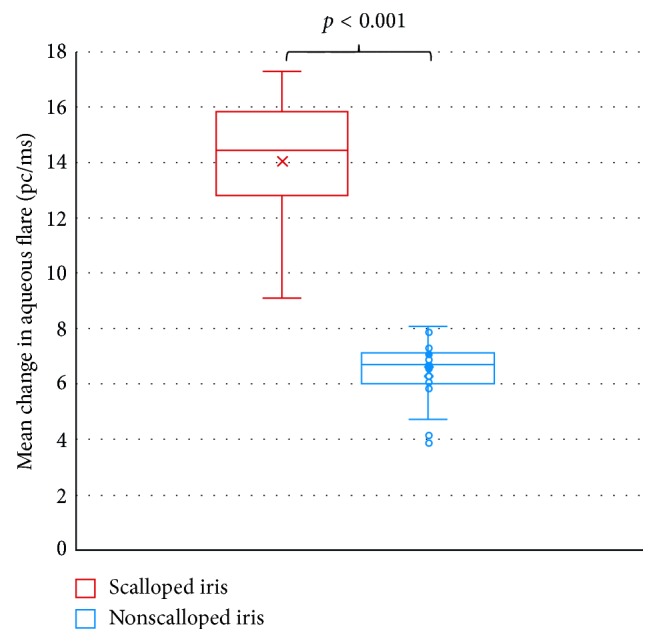
Aqueous flare distribution of ATTRVM30 patients with scalloped iris and nonscalloped iris eyes (control).

Fifty-six eyes of 28 ATTRV30M patients participated in this analysis. Patients' characteristics are shown in Figure 1 and Table 1. In this study, highly statistical significant differences were found in the disorder in the BAB between the scalloped iris eyes and the nonscalloped iris eyes (control). The aqueous humor flare values in the eyes with a scalloped iris were significantly higher than those in the nonscalloped iris control group (*p* < 0.001), while there were no significant differences in IOP between groups (*p*=0.163) (Table 2; Figures 2 and 3). There was no significant correlation between the flare and the IOP values within groups (scalloped iris eyes group, Spearman-rho = −0.06, *p*=0.749; nonscalloped iris eyes group (control), Spearman-rho = 0.28, *p*=0.135).

## 4. Discussion

The present study demonstrated that the aqueous humor flare values were significantly higher in the ATTRV30M scalloped iris eyes than those in the nonscalloped iris eyes (control group), and the IOP value was not significantly correlated with the increase in flare in both groups.

We previously reported a strong association between glaucoma and scalloped iris and advise to increase the frequency of IOP surveillance and look for glaucoma in these eyes [20, 28]. Nevertheless, our study showed no significant increase in IOP and na increased protein concentration in the aqueous humor in the scalloped iris eyes. These results may suggest the use of the flare meter method for predicting the risk of glaucoma in ATTRV30M scalloped iris eyes.

Several studies have addressed the changes to the aqueous humor proteome in glaucoma using other techniques with collection of aqueous humor samples after and/or during surgery [11, 13–19]. Inoue et al. reported higher levels of cytokines and growth factors in the aqueous humor of open-angle glaucoma eyes using multiplex bead immunoassay [13]. Imbalanced metabolism, lack of reactive oxygen species detoxification, low-grade, and chronic inflammation in the aqueous humor of open-angle glaucoma eyes were also assessed by several groups by mass spectrometry [15–21].

The laser flare meter is a precise, objective, and noninvasive method that can reliably measure the function of the BAB and the level of inflammation [25, 31]. The increase in aqueous flare echoes a disruption of the BAB, which allows leakage of serum proteins, as well as inflammatory molecules and cells, into the anterior segment by causing a change in aqueous protein composition and concentration [13, 26].

Evaluation of the anterior aqueous flare in patients with glaucoma has also been addressed recently [27–29, 32]. Many groups have assessed the anterior chamber aqueous flare in glaucomatous eyes after treatment with antiglaucomatous drugs. Interestingly, Kahloun et al. used the flare meter to evaluate the anterior chamber aqueous flare in patients with pseudoexfoliation syndrome with or without glaucoma and found that a high anterior aqueous flare could be a predictor for the development of glaucoma [27].

In our study, we observed an increased protein concentration in the aqueous humor of ATTRV30M patients with scalloped iris eyes without IOP increased values. An increase in protein concentration in the aqueous is the most straightforward evidence for the breakdown of the BAB [25].

Some authors hypothesize that the breakdown of BAB initially is due to endothelial injury caused by the mutant TTR [33, 34], but there must be another complementary mechanism since patients are transplanted and have no TTR mutant in circulation. The damage should be from outside the vessels were circulates the mutant TTR (aqueous humor and vitreous). As ATTR V30M patients have a proinflammatory state [35], we hypothesize that in response to this stimulation, the ciliary pigmented epithelium may release a large variety of cytokines and fibrin aggregates which may induce the breakdown of the BAB and outflow resistance in the anterior segment of the eye in ATTRV30M patients. As described previously, the intraocular production of TTR V30M by the retinal and ciliary pigment epithelium remains unchanged in these patients [8]. And the total aqueous humor TTR levels are almost the same in liver transplanted and nontransplanted ATTR V30M patients [10]. Thus, we hypothesize that an increased concentration of the unstable TTR V30M in patients' eyes could also contribute to the breakdown of the BAB and increased development of a mechanical barrier to the outflow of the aqueous humor, resulting in IOP elevation and worsening the prognostic for glaucoma development [30].

Therefore, anterior aqueous flare measurements should be considered in the staging and evaluation of the risk progression to glaucoma and be considered as a marker of ocular disease progression in ATTRV30M patients. The flare cell meter may help us to objectify the action of a possible treatment directed to the oculopathy and indirectly targeting the central nervous system [36, 37]. Further, longitudinal studies are warranted to assess the predictive value of aqueous flare for glaucoma development.

This study had some limitations. Further analysis is needed in order to determine which mediators are associated with the breakdown of the BAB function in these ATTRV30M patients. Furthermore, further histological or biochemical analysis to determine whether the source of increased aqueous flare values resulted from the breakdown of the BAB itself or simply from proteins diffusing from the posterior segment caused by the breakdown of the blood-retinal barrier.

## 5. Conclusions

In this study, we found that the aqueous humor flare value was significantly higher in the scalloped iris eyes than that in the nonscalloped eyes in ATTRV30M patients without significant differences in IOP between groups. As a marker for inflammation, and breakdown of the BAB, flare values suggest that controlling the stage and progression of glaucoma might be key in the surveillance scalloped iris eyes in ATTRV30M patients and may be considered as an evaluation method of future treatments.

## Figures and Tables

**Table 1:** Demographic and clinical data of ATTRV30M patients.

Characteristics	ATTRV30M patients (*n*=28)
Age, years	46.9 ± 5.6
Male/female	60.7%/39.3%
Time of ATTRV30M diagnosis, years	14.6 ± 3.9
Liver transplantation, %	96.4%
Time from liver transplantation, years	12.3 ± 3.4
Patients under tafamidis treatment, *n*	1

**Table 2:** IOP and aqueous flare values.

Parameters	Scalloped iris	Nonscalloped iris (control group)	*p* value
Mean flare (pc/ms)	14.1 ± 2.2	6.5 ± 0.9	*p* < 0.001
Mean IOP (mmHg)	17.1 ± 0.8	16.8 ± 0.7	*p*=0.163

## Data Availability

The data used in this study are available with the corresponding author. The access to data is restricted for legal and patient privacy reasons.
